# The Common Ancestor of Archaea and Eukarya Was Not an Archaeon

**DOI:** 10.1155/2013/372396

**Published:** 2013-11-17

**Authors:** Patrick Forterre

**Affiliations:** ^1^Institut Pasteur, 25 rue du Docteur Roux, 75015 Paris, France; ^2^Université Paris-Sud, Institut de Génétique et Microbiologie, CNRS UMR 8621, 91405 Orsay Cedex, France

## Abstract

It is often assumed that eukarya originated from archaea. This view has been recently supported by phylogenetic analyses in which eukarya are nested within archaea. Here, I argue that these analyses are not reliable, and I critically discuss archaeal ancestor scenarios, as well as fusion scenarios for the origin of eukaryotes. Based on recognized evolutionary trends toward reduction in archaea and toward complexity in eukarya, I suggest that their last common ancestor was more complex than modern archaea but simpler than modern eukaryotes (the bug in-between scenario). I propose that the ancestors of archaea (and bacteria) escaped protoeukaryotic predators by invading high temperature biotopes, triggering their reductive evolution toward the “prokaryotic” phenotype (the thermoreduction hypothesis). Intriguingly, whereas archaea and eukarya share many basic features at the molecular level, the archaeal mobilome resembles more the bacterial than the eukaryotic one. I suggest that selection of different parts of the ancestral virosphere at the onset of the three domains played a critical role in shaping their respective biology. Eukarya probably evolved toward complexity with the help of retroviruses and large DNA viruses, whereas similar selection pressure (thermoreduction) could explain why the archaeal and bacterial mobilomes somehow resemble each other.

## 1. Introduction

Archaea have been confused with bacteria, under the term prokaryotes, until their originality was finally recognized by 16S rRNA cataloguing [1]. Archaea were previously “hidden before our eyes”, strikingly resembling bacteria under the light and electron microscopes. Archaea and bacteria are also quite similar at the genomic level, with small circular genomes, compact gene organization, and functionally related genes organized into operons. At the same time, archaea, unlike bacteria, exhibit a lot of “eukaryotic features” at the molecular level [2–6]. It is often assumed that archaea resemble eukarya when their informational systems (DNA replication, transcription, and translation) are considered but resemble bacteria in terms of their operational systems. This is clearly not the case, since many archaeal operational systems (such as ATP production, protein secretion, cell division and vesicles formation, and protein modification machinery) also use proteins that have only eukaryotic homologues or that are more similar to their eukaryotic rather than to their bacterial homologues [7–14]. The bacterial-like features of some archaeal metabolic pathways could be mostly due to lateral gene transfer (LGT) of bacterial genes into Archaea, driven by their cohabitation in various biotopes [15]. Indeed, beside bacterial-like genes possibly recruited by LGT, metabolic pathways in archaea—such as the coenzyme A or the isoprenoid biosynthetic pathways—also involve a mixture of archaea-specific and eukaryotic-like enzymes [16–18]. Archaea and eukarya share so many features in all aspects of their cellular physiology and molecular fabric that eukaryotes cannot be simply envisioned as a mosaic of archaeal and bacterial features. Archaea and eukarya clearly share a more complex evolutionary relationship that remains to be understood.

Whereas many eukaryotic traits of archaea are ubiquitous or widely distributed in that domain, recent discoveries have identified several new eukaryotic traits that are only present in one phylum, one order, or even in one species of archaea [6, 11, 14]. Phylogenetic analyses suggest that these traits were already present in the last archaeal common ancestor (LACA) since, in most cases, archaeal and eukaryal sequences form two well separated monophyletic groups [12, 13, 19]. This indicates that these traits have not been sporadically acquired from eukarya by lateral gene transfer but were lost in most members of the archaeal domain after their divergence from LACA [6]. Considering this loss of eukaryotic traits and the gain of bacterial traits by LGT, LACA was probably even more “eukaryotic-like” than modern archaea. However, despite their eukaryotic affinity, archaea lack many eukaryote-specific features (ESFs) at the cellular and/or molecular levels. These, for example, include the spliceosome, mRNA capping, and extensive polyadenylation as well as huge transcriptional machineries with unique components, such as the mediator, endoplasmic reticulum, and derived structures such as lysosomes, the Golgi apparatus and the nuclear membrane, an elaborated cytoskeleton and associated vesicle trafficking system with endosomes and ectosomes, nuclear pores, nucleolus and other nucleus-specific structures, linear chromosomes with centromeres and telomeres, mitosis and associated chromosome segregation system linked to the cytoskeleton, complex and great sex with meiosis derived from mitosis, an incredible machinery for cell division apparatus with synaptonemal complex for meiosis, centrioles and midbodies for cell division, and I probably miss some of them. Archaea not only lack all these ESFs but also lack homologues of most proteins (a few hundreds) that are involved in building and operating them [20]. This remains true even if a few ex-ESPs (e.g., actin, tubulin, and DNA topoisomerase IB) have recently lost this status following the discovery of archaeal homologues [12, 13, 19]. The number, diversity, and complexity of ESFs are impressive and their origin remains a major evolutionary puzzle that should not be underestimated. The puzzle became of even greater magnitude when it was realized during the last decade from phylogenomic analyses that all ESFs (and associated ESPs) were most likely already present in the last common ancestor of all modern eukaryotes, (*the last eukaryotic common ancestor*) (LECA) [20]. In a recent review, Martijn and Ettema called the period that experienced the emergence of ESFs (so before LECA): *“the evolutionary dark ages of eukaryotic cells*” [21]. This denomination well illustrates the complexity of the “complexity problem” in eukaryotic evolution. 

Besides lacking (by definition) all ESFs, archaea also fundamentally differ from eukarya in the nature of their membranes (with a unique type of lipids in archaea), and the type of viruses infecting them. The problems raised by the evolution of membranes have been nicely reviewed recently by Lombard et al. and I will refer to their work later on to discuss different models for the origin of archaea [22]. In contrast, the problem raised by the drastic differences between archaeal and eukaryotic viruses has never been really discussed. For instance, Martijn and Ettema never mentioned the word virus in their review on the origin of eukaryotes [21]. Viruses are also completely absent from the papers of Cavalier-Smith or Carl Woese himself. This is probably because, as recently stated by Koonin and Wolf, “*viruses are no part of the traditional narrative of evolutionary biology*” [23]. 

## 2. The Bacterial Flavour of Archaeal Viruses and Plasmids: Another Evolutionary Puzzle

Viruses infecting archaea have fascinated for a long time scientists that are aware of their existence by the amazing morphologies of their virions that, in most cases, differ drastically from those produced by bacterioviruses (formerly bacteriophages) or eukaryoviruses [24, 25]. Among the 13–15 families of archeoviruses presently known, most are unique to archaea, and none of them is specifically related to a family of eukaryoviruses. The only archaeal viruses with eukaryovirus relatives are the archeoviruses STIV (*Sulfolobus islandicus* turreted virus) (see below) and Caudovirales, which belong to major lineages of viruses infecting members from the three cellular domains [26]. STIV is the archaeal member of the PRD1/adenovirus lineage that groups bacterial *Tectiviridae* (a group of small membrane-containing viruses resembling STIV) with large DNA viruses infecting eukaryotes, such as adenoviruses and Megavirales (formerly nucleocytoplasmic large DNA viruses, NCLDV) as well as the recently discovered satellite viruses (virophages) of giant Megavirales. Viruses of this lineage are characterized by major capsid proteins containing the so-called double jelly-roll fold. Archaeal and bacterial Caudovirales (head and tailed viruses) belong to the same viral lineage as eukaryoviruses of the family Herpesviridae. Their virions are constructed from the major capsid proteins displaying the so-called Hong-Kong 97 fold (structurally unrelated to the jelly-roll fold). Strikingly, the archaeal viruses in these two lineages are much more similar in virion size and overall structure to their bacterial than to their eukaryotic counterparts. In particular, archaeal and bacterial Caudovirales are identical in terms of virion morphology and genome organization and share several homologous proteins [27]. The three families of Caudovirales (Siphoviridae, Myoviridae, and Podoviridae) first described in bacteria have been now found in archaea [25, 27, 28]. Moreover, Caudovirales were recently found to be more widespread than previously thought among archaea, suggesting that Caudovirales already existed when archaea and bacteria started to diverge from each other [29]. Finally, a recently described family of archaeal pleomorphic viruses, pleolipoviruses, could be related to bacterial pleomorphic viruses of the family Plasmaviridae [30]. In summary, whereas archaea and eukarya share basic molecular biology features for all major ancestral cellular functions, the archaeal virosphere shares much more similarities with the bacterial one than with the eukaryotic one.

Beside common viruses, archaea and bacteria also share similar types of plasmids, insertion sequences (IS), and related transposons [25, 27–35]. In particular, Filée and coworkers were surprised by their observation that *“most of the archaeal ISs fall into family found in bacteria*” and that “*archaeal ISs resemble bacterial ISs rather than those identified in eukaryotes”* [31]. Furthermore, they detected no IS elements in archaeal genomes with significant similarity to the nine known superfamilies of eukaryotic DNA transposons [31]. Plasmids are abundant, diverse in size, and widespread in archaea, as in bacteria, contrasting with the paucity of plasmids in eukaryotes. Moreover, archaeal ISs and plasmids use bacterial-like proteins for transposition, plasmid resolution, and segregation [31]. Some of these proteins are only present in archaea and bacteria, and when they are universal (for instance initiator proteins for rolling circle replication) the archaeal version is more similar to the bacterial version than to the eukaryotic one [36, 37]. The bacterial affinity of archaeal viruses and plasmids confirms that these mobile elements are evolutionarily related, with plasmids probably being derived from ancient viral lineages [38]. Interestingly, viruses and plasmids encode many proteins that are involved in both bacterial and archaeal chromosome segregation and resolution, such as tyrosine recombinases of the XerCD/XerA family or ATPases of the ParA/SegA family [37, 39]. One can wonder if the similarity between archaeal and bacterial viruses/plasmids could explain the presence in many archaea of bacterial-like proteins involved in chromosome resolution/segregation. This would fit with a provocative scenario in which I suggested that the archaeal and bacterial chromosomes evolved from large DNA plasmids, with divergent replication mechanisms but homologous partition machineries, themselves derived from giant DNA viruses [40]. Finally, it is striking that archaea and bacteria use homologous defence systems against plasmids and viruses (CRISPR, toxin-antitoxin and restriction-modification systems) that are very divergent from the siRNA interference defence systems used by eukaryotes [41, 42]. Homologues of argonaute proteins, the core component of the eukaryotic interference system, have been detected in archaea and bacteria, but it is not yet known if these proteins are involved in an interference pathway [42, 43]. All these observations raise major unresolved questions: why so many archaeal mobile elements (head and tailed viruses and plasmids) are similar to bacterial ones, whereas archaea and eukarya are so similar in terms of molecular biology? Why, on the other hand, so many viruses infecting archaea are unique, having neither bacterial nor eukaryotic counterparts? A good theory for the origin of archaea and their relationships with eukarya should definitely explain these puzzling observations.

## 3. Different Scenarios for the Origin of Archaea and Eukarya

**Figure 1:**
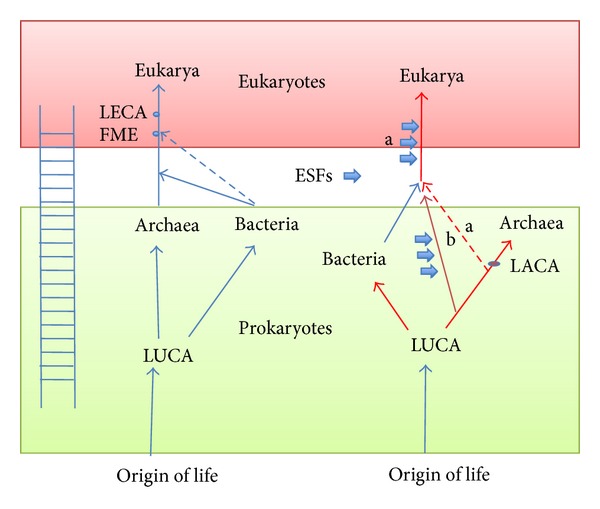
The Aristotle scenarios. (a) The traditional ladder-like evolutionary scenario, in which organisms increased in complexity from the origin of life to prokaryotes and eukaryotes, with archaea being intermediate organisms on the way to become eukaryotes; (b) the classical universal tree of life of Woese et al. [53] in red, combined with the fusion hypothesis (blue line). The LECA is the last eukaryotic common ancestor and FME the first eukaryote harbouring mitochondria; the dotted line refers to the hypothesis in which eukaryotes originated by the association of an archaeon with the mitochondrial bacterial ancestor [49]. Thick arrows indicate the emergence of eukaryotic specific features (ESFs).

Several scenarios are in competition to explain the origin of archaea and eukarya [20–22, 44–52]. The most popular presently are the fusion scenarios in which eukarya originated by the intimate association of an archaeon and a bacterium ([48, 49], reviewed in [46]; for a more recent hypothesis see [21]). In these scenarios, the fusion is triggered by the engulfment of one of the two partners (the endosymbiont) by the other (the host). This association is followed by a dramatic reorganization of the structures of the two partners (the fusion), promoting the emergence of a completely new type of cell (eukaryote instead of prokaryote). Several propositions have been made concerning the origin of the two partners (one archaeon and one bacterium) involved. The proposed scenarios also differ by the timing of the mitochondrial endosymbiosis. In some of them, this event takes place after the fusion [48], in others it corresponds to the fusion itself [49]. A common point to most fusion scenarios is that they involve two partners that are very similar to some modern archaea and bacteria [46]. These partners either belong to modern lineages of bacteria and archaea or are derived from an extinct (transient) archaeal lineage that originated from modern-looking archaea (as in the recently proposed *phagocytosing archaeon scenario* of Martijn and Ettema, [21]). This led to the common view that eukaryotes ascend from archaea in an evolutionary ladder *(scala natura*) leading from LUCA to eukarya via archaea (Figure 1(a)).

The fusion scenarios for the origin of eukaryotes are in apparent contrast to the classical tree of life proposed by Woese et al. in which archaea and eukarya are sister groups [53]. However, this tree can be reconciled with fusion scenarios if the archaeal-like partner diverged from the branch leading to archaea before the emergence of LACA. At some point. however, if this archaeal-like partner was very different from modern archaea (a protoarchaeon), with possibly additional eukaryotic traits, and if the bacterial partner was the alpha-proteobacterium at the origin of mitochondria, this “fusion hypothesis” becomes very similar to the “protoeukaryote” hypothesis in which the host of the alpha-proteobacterium was an organism belonging to a third lineage, distinct from lineages leading to archaea and bacteria [19, 50, 51, 57]. Accordingly, there is a continuum of possible scenarios between classical fusion hypotheses involving partners very similar to modern “prokaryotes” and the idea of a protoeukaryote “fusing” with the alpha-proteobacterial ancestor of mitochondria. In the following discussions, I will only refer to those fusion hypotheses that involve partners very similar to modern “prokaryotes.”

Beside fusion hypotheses, the classical “three-domain tree” has been challenged by scenarios in which the eukaryotic lineage emerges within archaea [55, 58–61]. To support this view, it has been repeatedly claimed recently that the three-domain tree should be replaced by an updated version of the “*eocyte tree*,” proposed by Lake et al. three decades ago [61] (eocyte being the name proposed by Lake et al. for the a group of thermophilic archaea, later on christened Crenarchaeota by Woese and colleagues [53]). The eocyte tree has been rejuvenated because it is apparently validated by phylogenetic analyses of universal proteins in which eukaryotes are nested within archaea [55, 58–60]. These analyses provided apparent support for a clade grouping eukarya with a candidate archaeal superphylum called TACK, that encompasses Thaumarchaeota, Aigiarchaeota, Crenarchaeota, and Korarchaeota [59]. Amazingly, if this is true, eukaryotes are members of the archaea (as we are apes) and should be considered as an archaeal phylum (and the TACK superphylum should be renamed TACKE since it also includes eukarya; otherwise, archaea would be paraphyletic, thus not a valid taxon, since the last common ancestor of archaea was also an ancestor of eukarya. 

Here, I will first briefly criticize the fusion scenario and argue in favour of the monophyly of archaea (so that we do not have to worry about TACKE). Then, I will discuss recent results that, in my opinion, strongly suggest that the last common ancestor of archaea and eukarya was more complex than archaea and less complex than eukarya (the “bug in between” scenario). I will call this ancestor thereafter the *last archaeal-eukaryal common ancestor* (LAECA). I will also try to put viruses in the general picture. 

## 4. Criticism of Fusion Scenarios

I have previously proposed my own fusion scenario, as a joke [46], accompanied by criticisms against my new version of the fusion (the association of a thaumarchaeon and a PVC bacterium). Since this paper is now often cited simply as a new fusion hypothesis (!), I have to reiterate here my antifusion arguments (for other critics, see [50–52]). In fusion scenarios, the host cell can be either a bacterium or an archaeon. In both cases, these scenarios first raise major problems for the fate of the ancestral membranes of the host or the endosymbiont, since the putative ancestral archaeal membrane has disappeared in eukaryotes. In all known real cases of symbiosis, the symbiont keeps its membrane, even in the most extreme cases of reduction (e.g., the mitoribosome or the nucleomorph) [62, 63] or when the symbiont uses lipids from their hosts, as in the case of *Nanoarchaeum equitans* [64]. Membrane conservation throughout evolutionary processes seems to be a major feature of cellular organisms [22]. This strongly contradicts with fusion hypotheses in which the host is a bacterium, since the archaeal membrane of the endosymbiont has completely disappeared after the fusion (the nuclear membrane in eukaryotic cells being derived from the bacterial-like membrane of the endoplasmic reticulum). In fact, disappearance of the membrane of an infectious entity is only known in the case of enveloped viruses, when the membrane of the viral particles (virions) disappears in the infected cell (i.e., fuses with the cellular membrane). However, this situation cannot be assimilated to a true endosymbiosis, since the virion is not an organism and should not be confused with the virus itself [65]. 

Scenarios with an archaeal host raise other problems, since they involve the transformation of the host archaeal membrane into the bacterial-like membrane of eukaryotes. Indeed, there is no obvious selection pressure that could have favoured this transformation. As noticed by Lombard and colleagues, such transformation has never been observed in nature [22]. In particular, acquisition of a bacterial type of membrane never occurred in any archaeal lineage, despite the massive lateral gene transfer (LGT) of bacterial genes into archaea. For instance, Nelson-Sathi and co-workers determined recently that about 1000 bacterial genes have been transferred, probably in a single event, in the ancestral archaeal lineage from which emerged all modern Haloarchaea [66]. Importantly, despite this massive transfer of bacterial genes, Haloarchaea still have “archaeal lipids” and remained *bona fide* archaea in terms of all their major cellular and molecular processes.

Fusion scenarios also involve highly unlikely *ad hoc* hypotheses to explain the emergence of all complex ESFs from the simpler compact and fully integrated molecular machines working in archaea and bacteria (for critics, see [44, 46, 50–52, 57]). There is no general agreement between proponents of fusion scenarios for most of these *ad hoc* hypotheses. For instance, several authors proposed different selection pressures to explain the origin of the eukaryotic nucleus [20, 67–69]. These hypotheses posit that (for various reasons) some kind of barrier was required between the nucleoid provided by one of the two partners and the cytoplasm provided by the other. However, they do not usually explain why all genes from the nucleoid of the host migrated through this barrier into the nucleoid of the endosymbiont (or *vice versa* depending on the scenario) and/or why the circular nucleoid of the endosymbiont/host was transformed into multiple linear chromosomes with telomeres and centromeres. They also do not explain why the simple “prokaryotic” cell division mechanism of the endosymbiont at the origin of the nucleus was replaced by the complex mitotic cell division machinery. 

Fundamentally, fusion scenarios posit that modern cells (archaea and bacteria) were transformed by their association into cells of a completely new domain (with an abrupt but transient acceleration of protein evolutionary rates leading to new versions of universal proteins in eukarya). This possibility was strongly rejected by Woese who wrote that: “*modern cells are sufficiently complex, integrated and ‘individualized' that further major change in their designs does not appear possible*” [70]. I fully agree with this statement; the observation of nature tells us indeed that such transformation is not possible. In all known cases of endosymbioses or close association between organisms that belong to different domains, both partners remain members of their respective domains and there is no dramatic acceleration of protein evolutionary rate, especially for universal proteins. For instance, the association between a cyanobacterium that produced chloroplasts did not transform Viridiplantae into a new domain. Viridiplantae remained eukaryotes (with eukaryotic ribosomes), whereas chloroplasts (and mitochondria) can still be recognized as highly derived bacteria, with highly divergent—but still bacterial—ribosomes. As already mentioned, the massive invasion of an Haloarchaeal ancestor by more than one thousand bacterial genes had no effect on the archaeal nature of Haloarchaea. 

Finally, another rarely discussed important argument against fusion scenarios is the uniqueness of eukaryotic viruses and related transposons [46]. Indeed, fusion scenarios posit that all modern eukaryotes originated from a unique fusion event between one particular archaeon and one particular bacterium. If the host was an archaeon, all eukaryotic viruses should have originated from those archaeal viruses that were able to specifically recognize the surface of this particular archaeon (or of its immediate descendants). Similarly, if the host was a bacterium, eukaryotic viruses should have originated from bacterial viruses that were able to recognize the surface of this particular bacterium (or its immediate descendants). This seems at odds with the present diversity of eukaryotic viral lineages and transposons, especially with the existence of many lineages of eukaryotic DNA and RNA viruses that have no viral counterparts in bacteria and eukarya, such as Baculoviridae, Megavirales, Retroviridae, and many others. One should posit that all eukaryotic viruses (in particular, most RNA viruses, retroviruses, and pararetroviruses) and transposons originated *de novo* after the fusion event, *in the dark age of eukaryotic evolution*, and/or that the ancestral archaeal or bacterial viruses and transposons evolved so fast after the mitochondrial endosymbiosis event that it is no longer possible today, with few exceptions, to recognize their evolutionary relationships with viruses and transposons infecting bacteria and archaea. These two possibilities seem unlikely. The *de novo* late origin of eukaryotic RNA viruses is at odds with the current assumption that RNA viruses are somehow relics of ancestral viruses from the RNA world. It is in particular appealing to think that ancestral retroviruses and/or retrotransposons played a key role in the transition from RNA to DNA genomes. The rapid and complete transformation of bacterial (or archaeal) viruses into eukaryotic ones (Caudovirales becoming Herpesviridae and *Tectiviridae*/STIV becoming Megavirales) and of archaeal/bacterial transposons into eukaryotic ones after the fusion event while archeoviruses and bacterioviruses, as well as bacterial and archaeal transposons, remained unchanged for billion years seems to me very unlikely.

In summary, fusion scenarios posit a transient but extreme acceleration of protein evolutionary rates and drastic structural changes to take into account the existence of eukaryotic specific versions of universal proteins (e.g., ribosomal proteins) and the rapid emergence of all ESFs in the period between the fusion event and LECA. They should also posit a transient but extreme acceleration of evolution of viral structures and the appearance of many new viral and transposon families in that same period. This does not seem reasonable, even more so if the fusion event is assimilated to the endosymbiosis that produced mitochondria [49], since in that case, all these dramatic evolutionary changes should have occurred between the appearance of the first mitochondrial eukaryote (FME) (i.e., after diversification of all bacterial lineages) and LECA! Such scenarios require no less than several miracles for the emergence of eukaryotes, miracles that occurred only once in 2-3 billion years of coexistence between archaea and bacteria. 

## 5. The Monophyly of Archaea

**Figure 2:**
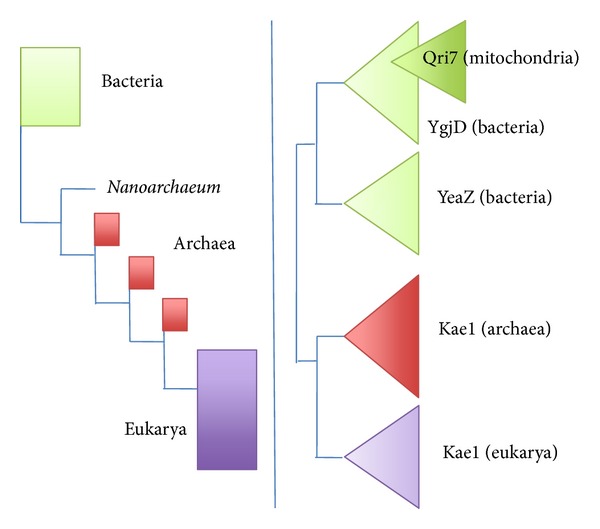
Two contrasting phylogenies of the same universal protein. Schematic representation of two published phylogenies of a universal protein known under different names (YgjD, YeaZ, Kae1/OSGEP, and Qri7/OSGEP L) which is involved in the biosynthesis of the universal tRNA modified base t6A [54]. These simplified phylogenies are adapted from Figure S46 in [55] (left panel) and from Figure S1 in [56] (right panel). Squares indicate unresolved nodes, whereas triangles indicate resolve nodes. The tree on the right is congruent with firmly established biological knowledge such as the monophyly of bacteria and eukarya, the bacterial origin of mitochondria. It favours the classical three-domain tree of Woese and colleagues. The tree on the left, which is not resolved, with aberrant paraphyly of archaea (see for instance the position of *Nanoarchaeum equitans*) was nevertheless used by the authors to support the “eocyte tree”. Comparison of these trees clearly reveals that the methodology (data sampling and/or algorithm for tree reconstruction) used by Cox and co-workers [55] for phylogenetic analyses cannot recover correct phylogenies for universal proteins.

**Figure 3:**
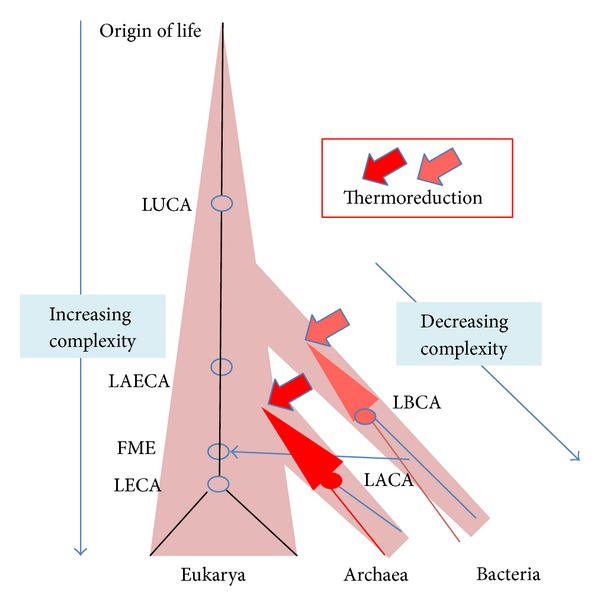
A scenario based on divergent evolutionary trends for “prokaryotes” (archaea and bacteria) and eukaryotes. This scheme is based on the assumption that the universal tree is rooted in the bacterial branch [53]. Complexity increased from the origin of life to LUCA to eukaryotes, via the last archaeal-eukaryal common ancestor (LAECA). Reductive evolution occurred from LAECA to LACA and modern archaea, possibly triggered by thermoreduction [77] indicated by large red triangles and/or as a way to escape protoeukaryotic predators [50]. Bacteria experienced independently a similar evolutionary path. The blue arrow indicates the mitochondrial endosymbiosis.

As previously mentioned, it is commonly assumed that the eocyte tree is now validated by phylogenetic analyses in which eukarya emerge from within archaea [55, 58–60], with the consequence that all eukaryotic ESFs should have originated in a highly divergent archaeal lineage and that archaea are our ancestors. However, these analyses, concerning very deep phylogenies, are prone to many artefacts (for a critical analysis of contradictory results obtained by different authors with more or less the same dataset; see [45]). In particular, phylogenetic analyses of Embley and colleagues [55, 58, 60] include many ribosomal proteins for which there is no significant signal for deep branching because bacterial proteins are too divergent from their archaeal and eukayotic homologues [71]. Elongation factors, amino-acyl tRNA synthetases, or else V-ATPases are also used in these analyses despite the fact that these proteins are heavily saturated with respect to amino acid substitutions [72] and cannot even resolve the phylogeny of eukarya, putting microsporidia (highly derived fungi) at the base of the eukaryotic tree. Several universal proteins used (RNA polymerases, RFC proteins and amino-acyl tRNA synthetases) are also encoded by many viruses (especially Megavirales) and it is unclear if the eukaryotic and archaeal versions are orthologues of if some of them have been independently acquired from viruses ([40, 73, 74]; see discussion below). The phylogeny of RNA polymerases is especially puzzling since the eukaryotic RNA polymerases of type I branch in between bacteria and a clade formed by archaeal and eukaryal RNA pol II and III [75]. In the analysis of Cox et al., the three homologous RNA polymerases are analyzed separately with their archaeal and bacterial homologues with RNA pol I being the only protein whose phylogeny supports the monophyly of archaea [55, Figure S27]. Strikingly, examination one by one of all phylogenies, published by Cox et al., shows that all of them failed to recover correctly the internal branching of the archaeal domain and are plagued with very bad resolution. In a more recent global analysis of a similar set of universal proteins, Lasek-Nesselquist and Gogarten [76] again obtained results favouring the eocyte tree, but they also notice that the method used *“generated trees with known defects, such as the placement of Microsporidia at the root of the eukaryotic tree, a paraphyletic Euryarchaeota, and an attraction of Nanoarchaeota to the base of the TACK + eukarya clade, revealing that this method is still error prone”.* Despite the exhaustive usage of complex alternative models to perform and test them, the phylogenies used in these global analyses cannot provide answer to the question of archaeal monophyly *versus* paraphyly because, in most cases they lack valid phylogenetic signal. Moreover, the quality of these phylogenetic analyses itself is also questionable. To be convinced, compare the phylogenies of the protein YgjD/Kae1 (involved in a universal tRNA modification) published by Cox and co-workers, with those published in Hecker et al. in [56] (Figure S46 in [55], the YgjD/Kae1 protein is named O-sialoglycoprotein endopeptidase, according to an ancient annotation that turned out to be wrong [56]) (Figure 2). In the tree of Cox and co-workers the phylogenies of the bacterial and eukaryotic proteins are not resolved, and the archaea are paraphyletic, with eukarya branching with *Methanopyrus kandleri* and Crenarchaeota! In striking contrast, the phylogeny of all domains is well resolved in the tree published in Hecker et al., and the overall phylogeny exhibits a clear-cut three-domain topology [56]. However, the three can be divided in to five groups because an ancient duplication occurred in the bacterial domain leading to two paralogous proteins, YgjD and YeaZ, and mitochondrial proteins, named Qri7, branch within the YgjD tree, in agreement with their bacterial origin (Figure 3). The surprisingly unresolved YgjD/Kae1 phylogeny published by Embley and colleagues suggests that their analyses favouring the paraphyly of archaea, with the emergence of eukarya within archaea, correspond to the concatenation of poorly resolved phylogenies and are plagued by multiple methodological problems.

Some logical considerations argue in fact against the emergence of eukarya within archaea (thereafter called the “*archaeal ancestor scenario”*). In that scenario, the archaeal ancestor should have contained all eukaryotic features that are presently dispersed in modern archaea. In that case, since LACA was probably more eukaryotic-like than any one of its descendants [6], the archaeal ancestor should have been LACA itself or a descendant of LACA, which, unlike the others, never lost a single eukaryotic feature. At this point one should remind that LACA was not a special (breakthrough) organism but simply the *last* of all common archaeal ancestors that thrived between LUCA and LACA. Why this particular ancestor should have also been the ancestor of eukarya? Another complication for the archaeal ancestor scenario is that LACA was probably a hyperthermophile [6, 80, 81]. The only mesophilic organisms presently known in the putative TACKE phylum are mesophilic Thaumarchaeota [82]. However, all known mesophilic Thaumarchaeota lack some critical eukaryotic features, such as actin or tubulin [6]. Again, one should suppose that eukarya originated from a mesophilic descendant of LACA, which has never lost a single eukaryotic feature and left no other descendants itself besides eukarya! Finally, the archaeal ancestor scenario raises the same problems as those of the fusion/association theory concerning (1) the transformation of the archaeal membrane into the eukaryotic one, (2) the deconstruction of the very efficient and integrated prokaryotic-like molecular biology of archaea, such as the coupling of transcription and translation, into the complex and often odd eukaryotic molecular biology, and (3) the origin of eukaryotic viruses and transposons, which, in that model, should have all originated from evolutionary unrelated archaeal viruses and transposons (!) or originated recently, *de novo*, in *the dark age of eukaryotic evolution*. 

## 6. Divergent Evolutionary Trends Shaped the History of Archaea and Eukarya

We have always the tendency to interpret evolution as a general trend from simple to complex because, as *Homo sapiens*, we are still under the spell of the Aristotle's *scala natura*. This is why the idea that human originated from apes looking like chimps was so prevalent in narratives describing our origin. However, it seems now that our common ancestor with chimps was possibly already bipedal, looking more like an ancient *Homo* than a modern chimp [83]. I will bet here that, as our last common ancestor with chimps neither resembled chimps nor *Homo*, LAECA** **was neither an archaeon nor a eukaryote, but a creature endowed with the property to be at the origin of both (Figure 3). To draw a picture of LAECA, I will use as Ariadne's thread the concept of evolutionary trends, knowing that these trends can be different for different lineages, depending on their ecological setting and “ways of life”; that is, whereas in some lineages organisms become more and more complex** **by the acquisition of novel traits and the use of more components to perform the same task, in other lineages, they evolve by reduction (streamlining), by losing ancestral traits and simplifying molecular processes [84].

It has been clearly shown recently from comparative genomics that a long suspected trend toward simplification indeed occurs in archaea and bacteria. For instance, Wolf and co-workers have shown that gene losses are estimated to outnumber gene gains at least 4 to 1 in these two domains [85]. Importantly, two independent studies concluded that LACA was an organism of greater complexity than most of the extant archaea [85, 86], in agreement with the observation that the ancestral archaeal ribosome contained more proteins than the ribosomes of modern archaea [87, 88] and that LACA should have combined all eukaryotic traits presently dispersed in various archaea [6]. Phylogenomic analyses focusing on protein structures also detected a trend toward proteome reduction in the archaeal and bacterial lineages, suggesting that both lineages originated from ancestors with more complex proteomes ([89–91] and references therein). All these observations are in agreement with ancient ideas proposed by scientists like Carlile who argued long ago that “prokaryotes” with their highly integrated and efficient molecular biology have evolved by streamlining to increase their reproduction rate and use of resources in rapidly changing environments (r-selection) [92].** **There are of course some exceptions to this general trend, especially in bacteria, such as the late evolution of giant bacteria in subgroups of proteobacteria [93], but these are the exceptions that confirm the rule. 

In contrast to the overall trend toward simplification observed in archaea and bacteria, an evolutionary trend toward more complex forms, slower growth rates, and larger size is clearly operating in eukaryotes [92]. This trend can be seen of course in animals, with the emergence of the immune and nervous systems and finally brains becoming larger and larger in some lineages, but it can be also observed in plants and fungi, which now rule the macroscopic world and in various protists with primary and secondary endosymbioses producing very sophisticated organisms. The evolutionary trend toward complexity in eukaryotes is very ancient since LECA was already as complex as modern eukaryotes in terms of cellular structure and molecular biology, with, in particular, a genome full of introns [94]. This trend toward complexity is again only an overall trend and reductive evolution in eukaryotes led, for instance, several times independently to the transformation of multicellular fungi into unicellular yeasts [95]. 

I will thus argue here that LAECA was not an archaeon as is currently assumed, but an organism from which two lineages, whose destiny was shaped by opposite evolutionary forces, have diverged. One led to the emergence of archaea by reduction, the other to eukarya by increasing complexity (Figure 3). Indeed, if evolution by reduction has taken place from LAECA to modern archaea and increasing complexity from LECA to modern eukarya, there is no reason to imagine that these respective trends became effective only at the time of LACA and LECA, respectively. LACA and LECA were not special organisms on the two evolutionary lines that can be drawn from LAECA to modern organisms (Figure 3). They are only the most recent organisms that all archaea or eukaryotes share as common ancestors, respectively (much like the African Eve and Adam who were not special individuals in the *Homo sapiens* lineage but only those at the origin, respectively, of all women—for Eve—and men—for Adam—living today). 

If the two opposite evolutionary trends discussed above are related to ancient differences in the way of life that have very early on fashioned cell structure and function in the two respective lineages, there is therefore no obvious reason why these trends should have changed at the time of LACA and LECA. A weak point of the archaeal ancestor hypothesis is that it precisely involves a dramatic reversal of the reductive trends at work in archaea, as if a particular lineage of yeast started today to evolve back toward extremely complex fungi. As previously discussed, to posit that this trend reversal was triggered by the endosymbiosis of a bacterium is at odds with current observations showing that endosymbioses never modify the basic molecular biology of the host and usually follow a previous evolutionary trend of the host toward complexity in order to capture the symbiont. This is even recognized by recent proponent of fusion models, which now imagine that the host was an archaeon more complex than modern ones, with already elaborated phagocytic capacities, as in the “*phagocytosing archaeon theory”* of Martijn and Ettema [21]. It seems to me more logical to think that eukaryotes did not evolve from a particular archaeal lineage, which was at odds with the archaeal evolutionary trend, but from a LAECA that was more complex than LACA, but less complex than LECA. LAECA was definitely not an archaeon since, by definition, all archaea have originated from LACA, whereas in the scenario proposed here, LACA itself originated from LAECA.

## 7. The Origin of Archaea: The Thermoreduction Hypothesis

**Figure 4:**
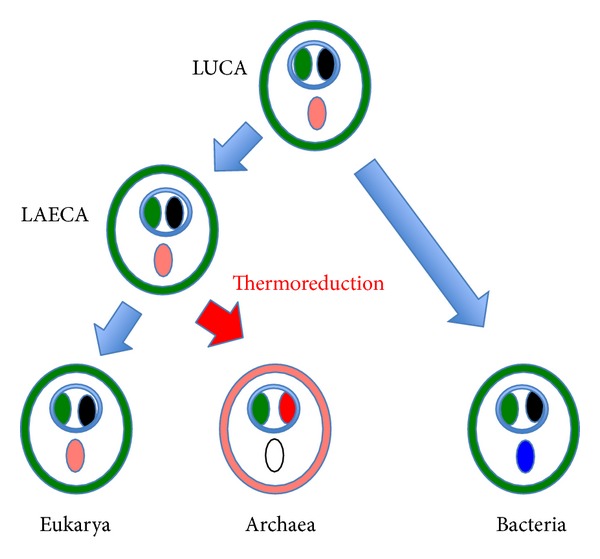
Schematic evolution of cellular membranes.** **Distribution of proteins involved in phospholipid biosynthesis in the three domains, LUCA, and LACA are depicted according to Lombard et al., [18, 22], but my evolutionary interpretation is different from those proposed by these authors [22]. Bacterial/eukaryal type membranes are in green and archaeal type membrane in red. Green circles correspond to universal enzymes involved in phospholipid biosynthesis (glycerol phosphate dehydrogenases, enzymes linking polar head groups to glycerol). Pink circles correspond to the classical mevalonate pathway for isoprenoid biosynthesis that was probably present in LUCA and lost in bacteria. Red circle represents enzymes of the alternative mevalonate pathway, which are specific of archaea, and involves a mixture of eukaryotic-like and archaeal specific enzymes. Blue circles correspond to the nonhomologous methylerythritol pathway for isoprenoid biosynthesis present in bacteria. Black circles represent the fatty acid biosynthetic pathway, which is no longer used for membrane phospholipid biosynthesis in archaea. Circles corresponding to proteins involved in the biosynthesis of membrane phospholipids are encircled.

Gouy and his co-workers have shown, using ancestral rRNA and universal protein sequence reconstruction, that LUCA was probably a mesophile, whereas LACA was probably a hyperthermophile (i.e., an organism living at temperature above 80°C, [96]) [80, 81]. Since all known eukaryotes are mesophiles, it is thus more parsimonious to think that LAECA itself lived at moderate temperature, the transition from mesophile to thermophily having taking place between LAECA and LACA (Figure 3). This would provide in particular a highly plausible selection pressure to explain the emergence of the archaeal membrane [97–99]. The unique chemical and structural features of archaeal lipids are indeed perfectly suited to maintain the cytoplasmic membrane functional at high temperature [100]. In particular, the archaeal membrane is much less permeable to protons/ions than the bacterial and eukaryotic one [100]. This last point is critical for the maintenance at high temperature of the transmembrane gradient of ions/protons required for ATP synthesis. Interestingly, phylogenomic analyses strongly suggest that many enzymes involved in the biosynthesis of both bacterial and archaeal building blocks for lipid biosynthesis were already present in LUCA [18, 22]. Lombard and colleagues thus suggest that LUCA had already both types of lipids, having a mixed archaeal/bacterial membrane. However, modern cells also contain much of these enzymes but use a single type of membrane, either an archaeal one for archaea or a “bacterial one” for bacteria and eukarya. The building blocks, such as isoprenoids, used in Archaea for the synthesis of membrane lipids, are used for other tasks in bacteria and eukarya, and *vice versa* [18, 22]. If LAECA was a mesophile, it thus makes sense to imagine that it possessed the “bacterial type” of lipids that are still present today in eukarya and that archaeal lipids emerged specifically in the archaeal lineage under the pressure of adaptation to higher temperatures (Figure 4). In agreement with this view, Lombard et al. have shown that Archaea and eukarya share unique enzymes involved in the synthesis of archaeal lipids that differ from the bacterial ones, but that archaea also use a few archaea-specific enzymes required for the biosynthesis of archaeal membranes [18, 22]. This indicates that important modifications occurred specifically in the archaeal lineage, from LAECA to LACA, and it seems reasonable to suggest that these modifications are those that were involved in the formation of the unique archaeal type of membrane, perfectly suited for life at high temperature (Figure 4). 

More generally, the selective pressure behind the trend toward reduction from LAECA to LACA might have been the progressive adaptation of the archaeal ancestors to hotter environments in the framework of the “*thermoreduction hypothesis*” for the origin of “prokaryotes” [77] (Figure 3). The major features of the “prokaryotic” phenotype (coupling of transcription and translation, short half-life of messenger RNAs, small cell, and genome sizes, and high macromolecular turnover) are indeed perfectly suited for life at high temperature [77]. This could explain why the upper temperature limit for eukaryotes, around 60°C, established by Brock in the sixties [101] has never been exceeded from that time, despite the extensive search of hyperthermophilic eukaryotes that ended up with thermophilic protists growing up to 54°C [102]. The adaptation of archaeal ancestors to high temperature might also explain why they got rid of RNA viruses that possibly infected LAECA and still infect eukaryotes, since RNA is very unstable at high temperature [77, 97], making it difficult to imagine RNA genomes remaining intact for long periods in virions bathing in hot ponds. Bolduc and co-workers identified by PCR putative novel positive strand RNA viruses in an archaea-rich hot springs [103], but the archaeal nature of their host cells remains to be demonstrated. In fact, the “thermoreduction hypothesis” that I proposed 20 years ago is now strongly supported by the more recent work of Gouy and colleagues on the reconstructed temperatures of LUCA and LAECA [80, 81] and well explains the evolutionary trend toward reduction now recognized in the archaeal domain [85, 87], suggesting that one can probably extrapolate this trend from LACA back to LAECA. The thermoreduction hypothesis can be also coupled to the “raptor scenario” proposed by Kurland and colleagues, since the possibility to explore hot environments may have been a formidable advantage for prokaryotes in hiding themselves from protoeukaryotic raptors unable to follow them to hell [46].

## 8. The Mechanisms of Eukaryogenesis

We have seen that LECA was already a very complex organism already endowed with all ESFs of modern eukaryotes [20, 104]. Part of this complexity might have been due to the acquisition of mitochondria that allowed a dramatic input in energy available for macromolecular biosynthesis [105]. LECA was thus certainly more complex than the first mitochondrial eukaryote (the FME), that is, the organism harbouring the alpha-proteobacterium at the origin of modern mitochondria (thereafter called the mitochondrial ancestor). It has been shown for instance that the mitochondrial ribosome of LECA has already acquired 19 new proteins of unknown origin (and lost one bacterial protein) compared to the FME [88]. 

Lane and Martin [105] have argued that the energy produced by mitochondria with their core genome encoding proteins of the respiratory electron transport chain was essential for the emergence of eukaryotic complexity, including the emergence of all ESFs. However, eukaryotes that have lost mitochondrial genomes have not changed their eukaryotic fabric and still harbour most, if not all, ESFs. This suggests that EFS may have appeared before the mitochondrial-driven increase in cellular and genome sizes typical of large complex eukaryotes. The idea that all ESFs originated in the time period from the FME to LECA also implies that the bacterial ancestor of mitochondria was not captured by phagocytosis. However, the presence of an elaborated cytoskeleton and machinery for vesicle formation (exosomes, ectosomes, endosomes, etc.) in the FME seems also logical, considering that actin, tubulin, and G-ATPases were already present in LAECA. LUCA itself might have been compartmentalized, considering the structural similarities detected between eukaryotic coat proteins (involved among other things in nuclear pore formation) and proteins present in compartmentalized bacteria of the superphylum PVC (Planctomycetales, Verrucomicrobiales, and Chlamydiae) [106]. In that case, it is logical to think that the mitochondrial ancestor was obtained by phagocytosis, as it was probably the case later on for the ancestor of chloroplast, and as it is still now for so many intracellular bacteria living in eukaryotic cells. Beside phagocytic capacities, I suggest here that most ESFs were already present in the FME, even if some of them might have been profoundly altered following the assimilation of the mitochondrial ancestor. This is in agreement with the idea that the FME itself was more complex than LAECA because the evolutionary trend toward higher complexity was already operational during the period between LAECA and LECA (via the FME) (Figure 3).

The mechanisms that induced increasing complexity in the eukaryotic lineage before LECA can be inferred from comparative genomics and from the analysis of the mechanisms involved in recent complexity increase in modern eukaryotes. Eukaryotes typically use multiple paralogous proteins to build complexes that are composed of a single (or very few) paralogous protein in archaea. For instance the archaeal MCM helicase is a homohexamer, whereas the eukaryotic MCM helicase is a heterohexamer made of six paralogous MCM2-7 proteins. Eukaryotes use three paralogous RNA polymerases (I, II, and III) for transcription, whereas archaea use only one. Eukaryotes possess five DNA polymerases of the B family *versus* 1, 2, or 3 in archaea. 

**Figure 5:**
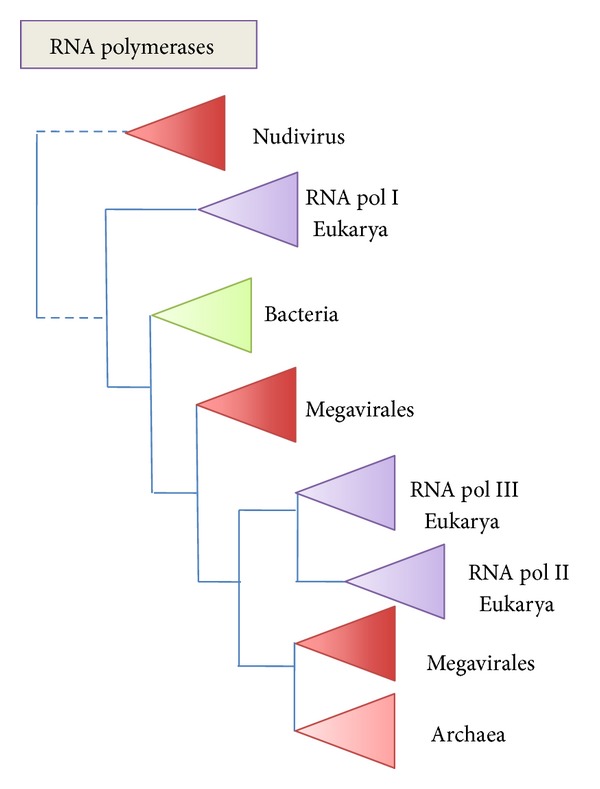
Schematic phylogenies of RNA polymerases. The RNA polymerase tree has been drawn combining and interpreting results from several papers [54, 60, 78, 79]. The position of the Nudivirus RNA polymerase is extrapolated from its high divergence with other homologous RNA polymerases (indicated by dotted lines). An update phylogeny would be welcome but would not change the take-home message, the puzzling mixture of cellular and viral enzymes, suggesting several ancient transfers between viruses and cells.

The complexity of eukaryotic molecular mechanisms compared to their archaeal homologues has been attributed to extensive gene duplications from LAECA to LECA that roughly double the number of genes in eukaryotic core genomes [20]. Indeed, gene duplications, and even whole genome duplications, have been involved in more recent stages of eukaryotic evolution. Here, however, I would remind that the multiple “paralogous proteins” present in the ancient core genomes of eukaryotes might not be all true paralogues, but in some cases homologues introduced by viral integration into the genomes of protoeukaryotes [46, 74]. This phenomenon has been observed in the case of the evolution of the archaeal DNA replication apparatus. For instance, it was deduced from phylogenetic analyses that 4 out of 6 MCM genes present in the genomes of some Methanococcales were recruited from mobile genetic elements (viruses or plasmids) [107]. Many eukaryotic viruses with large DNA genomes encode transcription or replication proteins homologous to eukaryotic ones and, via integration in the genomes of protoeukaryotes, their ancestors might have been the source of multiple different versions of homologous genes in modern eukaryotic genomes [46]. This would explain for instance the odd phylogenies of eukaryotic RNA or DNA polymerases (see Figure 5 for a schematic tree of RNA polymerases) [75, 108]. In these phylogenies, the multiple versions of the eukaryotic enzymes do not form monophyletic groups, themselves sister groups of their archaeal homologues, as would be expected if they originated by gene duplication in eukaryotes after the divergence of archaea and eukarya. Instead, they are paraphyletic, often nested with enzymes encoded by DNA viruses, and they are intermixed with archaeal enzymes. 

Ancestors of Megavirales could have been a major source of new genes in the eukaryotic linage [46]. These viruses, with genome sizes varying from 150 kb to more than 1 Mb, are very ancient and most likely predated LECA [109]. Moreover, integration of Megavirales genomes into eukaryotic genomes has been documented [110]. The abundance of lineage-specific proteins in the various lineages of Megavirales testifies for the genetic creativity of these viruses [111], indicating that they might have been an important source of new genes both before and after LECA. The integration of viral genomes thus provides the hosts with new proteins that can acquire important functions. There are many examples in the evolution of modern eukaryotes that testify for the importance of viral proteins in the evolution of eukaryotes. For instance, exaptation of a retroviral protein, syncytin, has been critical for the formation of placenta in mammals [112]. These phenomena also occur in archaea and bacteria, but considering the extreme abundance of mobile elements in eukaryotic genomes, their importance in eukaryotes is an order of magnitude higher.

The integration of ancient Megavirales can also explain why the eukaryotic core genomes contain many bacterial genes of different origin and without affinity to the mitochondrial alpha-proteobacterium ancestor. These bacterial genes are usually supposed by proponents of fusion hypotheses to derive from the bacterial partner [113–117]. Alternatively (or in addition) these genes are supposed to be derived from genes present in bacteria that were eaten by protoeukaryotes (“*you are what you eat*”, [118]) or in bacterial endosymbionts of ancestral eukaryotes [20]. I suggest here that many of these genes have not been directly transferred from bacteria to protoeukaryotes but that these transfers have been mediated by ancient Megavirales. Remarkably, these giant viruses contain today up to 10% of bacterial genes in their genomes [119]. Megavirales with linear genomes have probably recruited these genes from endosymbiotic bacteria living in their hosts via their specific DNA replication mechanism [119]. Bacterial genes are also present in Megavirales with circular genomes, indicating that linear genomes (typical of eukaryotes) could have preceded circular ones (typical of “prokaryotes”) in genome evolution [74]. Eukaryotes might have also recruited their messenger RNA capping system from ancient Megavirales [120], since these viruses use for capping the eukaryotic system, whereas other lineages of viruses use different systems [121]. The viral diversity in terms of capping mechanisms suggests that different capping mechanisms originated several times independently in the viral world and that one of them was transferred later on into the protoeukaryotic lineage [120]. The idea to tag chemically its own messenger RNA to discriminate it from the messenger RNA of your host can be viewed as a typical viral trick that was later on stolen by eukarya in the arms race between viruses and cells. 

Besides, megavirales, retroviruses, retroelements, and other eukaryotic transposons have been probably a major source of variations at the origin of eukaryote evolution [46, 74]. These genetic elements, which represent a large portion of modern eukaryotic genomes, have been critical factors in recent eukaryotic evolution [122]. The mobility of IS triggers rapid genome rearrangements and modifies genome expression patterns, providing new promoter elements, activating or regulating genes, even creating new genes by interfering with alternative splicing. It is therefore tempting to suggest that retroviruses have been used as toolkits in the formation of some ESFs at the onset of the eukaryotic lineage. 

Retroviruses and/or derived retroelements have been probably instrumental in the emergence of eukaryotic chromosomes. Indeed, telomeres are evolutionarily related to retroposons of the Penelope family [123, 124] and telomerases are homologous to reverse transcriptases [125]. Moreover, centromeres are formed by the repetition of numerous retroelements [126]. The cell nucleus itself might have originated as a protective device allowing the cell to hide their chromosomes from viruses [46, 120, 127]. For this purpose, cells might have recruited viral proteins able to manipulate the endoplasmic reticulum membranes, and that were originally used to build viral factories [120]. This scenario is supported by the fact that viruses themselves use viral factories to protect their replication machinery from the defence systems of the host [128]. Protoeukaryotic cells might have learned from viruses how to use this trick against them by protecting their genomes from viral attack, building giant “cellular factories” that became cell nucleus. It is indeed remarkable that the “volcano-like” viral factories of giant Megavirales, such as *Mimivirus*, are in fact as big as the large nucleus of their amoebal host cells [129]. 

I have previously emphasized the profound difference existing between the eukaryotic defence mechanisms against viruses and those used in common by archaea and bacteria. The arms race between eukaryotes and their specific viruses or else the necessity for eukaryotic cells to somehow control the spread of their specific transposons probably played an immense role in the specific evolution of the eukaryotic cells. It is clear for instance that exaptation of the siRNA antiviral defence system by eukarya to produce various types of microRNA has been decisive in the evolution of eukaryotic cells toward complexity. Modern eukaryotes use DNA methylation and histone modifications to limit the spread of mobile elements [122]. It is possible that these mechanisms that are typical of eukaryotic cells first originated as tools in the arms race between eukaryotes and their viruses and were recruited only later on for gene regulation in eukaryotes. 

I think that it is highly significant that Megavirales, retroviruses, and transposons with their associated defence/attack systems, which have been probably essential for the evolution of eukaryotes toward complexity, are missing in archaea and bacteria. Interestingly, these megavirales and retroviruses are also unknown in yeasts. One can wonder if the loss of most viral families in yeasts, especially retroviruses, could explain why these unicellular eukaryotes seem to be trapped in their “prokaryotic lifestyle” and have never evolved back toward complexity. The absence of the siRNA antiviral defence system in archaea and bacteria is also remarkable. For me, the lack of these features could explain why these “prokaryotes” did not evolve toward greater complexity, even when many of them finally escaped the selection pressure toward streamlining linked to thermoreduction. When archaea and bacteria finally invaded mesophilic biotopes, they could not anymore “benefit” from the evolutionary toolkit for increasing complexity provided in eukaryotes by retroviruses, retroelements, and megavirales.

## 9. A Picture of LAECA

If we agree that LAECA was more complex than archaea and less complex than eukarya, the main question now is how much was it more and less complex than archaea and eukarya, respectively? In one extreme view, close to the *archaeal ancestor* scenario, the period between LAECA and LACA was short and LAECA was only slightly more complex than LACA (the “prokaryotic LACA). In that scenario, thermoreduction played only a minor role in shaping LACA, and all ESFs originated from LAECA to LECA. At the other extreme, most ESFs were already present in LAECA (the “eukaryotic” LAECA) and lost from LAECA to LACA. I suspect that the truth is again “somewhere in between”. However, many evolutionists traditionally favour the “prokaryotic LACA” view. To counterbalance, I will try to push somewhat in the other direction. It is now often assumed for instance that the spliceosome machinery of eukarya derived from group II introns present in the mitochondrial ancestor or in ancient endosymbiotic bacteria, because some RNA components of the spliceosomes are evolutionarily related to group II introns [67, 68, 130]. However, this does not explain why intermediates of this evolutionary process have never been observed, despite the fact that eukaryotes have continued to coexist after LECA for 1-2 billion years with intracellular bacteria harbouring group II introns. Moreover, comparative genomics analyses have shown that the LECA had a spliceosome and contained probably a plethora of spliceosomal introns [94] and it seems unlikely that such incredibly complex molecular machine could have emerged in a short time between the FME and LECA. Interestingly, it has been shown recently that the genomes of some nucleomorphs have lost all introns and all genes encoding components of the spliceosomal machinery [131]. The nucleomorphs are remnants of eukaryotic nucleus from eukaryotic endosymbionts that are present in some photosynthetic protists. This observation tells us that the spliceosome (and protein coding genes containing introns) could be in fact an ancestral feature that was completely lost in bacteria and eukarya, while being retained in eukarya. This scenario would make sense since the spliceosome machinery (a giant ribozyme) reminds strikingly the ribosome, suggesting that both are remnants of the RNA world. 

Kurland et al. have suggested a few years ago that the “prokaryote” ancestors evolved by streamlining to escape protoeukaryote praying on them by phagocytosis (the phagotrophic unicellular raptor scenario, [50]). This would imply the presence of an already quite elaborated cytoskeleton in LAECA, with possibly already an endoplasmic reticulum. 

I have no space here to discuss the timing of appearance of the modern eukaryotic nucleus with typical eukaryotic chromosomes, mitosis and meiosis, and so on. I will argue that we should try to answer the question of their presence/absence in LAECA, by thinking in terms of reversibility or irreversibility. Is it possible or not for a specific ancestral ESF, once established, to have completely disappeared in archaea and bacteria? The nucleomorph story tells us that the answer is yes for the spliceosome. Beside intellectual constructions, we should look for similar examples to try obtaining answers for other ESFs. 

A major question whose answer could help us to go further in our scenario is, what kind of viruses infected LAECA? If viruses are very ancient, as now suspected, having emerged well before LUCA [26, 132, 133], the logical conclusion would be that LAECA and closely related organisms living at that time were infected by ancestors of all viruses infecting now archaea and eukaryotes. This would mean that protoretroviruses and protomegavirales were around at that time and have been later on lost in the archaeal lineage. If true, as previously discussed, this might have maintained this lineage irreversibly into the path of reduction. Major questions then remain to be tackled such as, why so many lineages of archaeal viruses, such as Fuselloviridae, Rudiviridae, Lipothrixviridae, Clavaviridae, and so on, have disappeared in eukarya? One possibility may be that invention of the nucleus dramatically reduced the number of viral families capable to survive this invention, because, originally, only a few viruses were able to replicate in the cytoplasm. In that case, this would mean that the nucleus originated after LAECA. One can also wonder why eukarya lost the CRISPR system. This system was possibly more specific to those viruses that were lost in eukaryotes? Similarly, one can wonder why archaea lost the siRNA interference system? A reasonable possibility is that the *raison d'être* of this system, to detect and kill RNA viruses by targeting their genomes, disappeared once “protoarchaea” got rid of RNA viruses. Interestingly, many viral families known in Crenarchaeota, such as Rudiviridae, Lipothrixviridae, and Clavaviridae, are presently unknown in Euryarchaeota. It seems unlikely that these viruses originated *de novo*, in the branch leading to Crenarchaeota. If these viruses were present at the time of LACA, this implies that these vital families were eliminated in the lineage leading to Euryarchaeota. If this is the case (much more work on archaeal viruses will be required to confirm this scenario) this could indicate that loss of viral lineages is indeed a common feature in the emergence of novel cellular lineages.

## 10. The Path from LUCA to LAECA

I was critical of the traditional rooting of the tree of life in the bacterial branch, because this rooting is not supported by robust phylogenies and is often interpreted as supporting a “prokaryotic” LUCA [134, 135]. These previous observations remain valid but let open the resolution of the rooting problem itself. I used to favour the idea that the more complex molecular features observed in archaea/eukarya compared to archaea (e.g., more RNA polymerase subunits) were possibly ancestral. However, I am changing my mind because scenarios in which all specific archaea/eukaryotic proteins were present in LUCA and replaced later on systematically by the bacterial version seem more and more unlikely with increasing knowledge on the molecular mechanisms involved. For example, nonhomologous archaeal (eukaryal) and bacterial ribosomal proteins or transcription factors often occupy the same site on the ribosome and RNA polymerase, respectively, and it is difficult to imagine how one set of proteins was replaced by the other. It seems more likely that, once established, the bacterial and the archaea/eukaryotic versions of molecular machines could not have changed drastically. In agreement with this view, these machines then remained similar in all lineages that diverged from the ancestors of bacteria and archaea/eukarya, respectively. These considerations argue in favour of a relatively “simple” LUCA, as originally suggested by Woese and Fox under the name progenote [136], possibly still a member of a cellular RNA world [137]. In that scenario, LAECA was certainly more complex than LUCA, since the complex archaeal/eukaryotic versions of molecular system were now all present in that organism (Figure 3). I previously suggested that LAECA had possibly still an RNA genome to explain some major differences between the DNA replication apparatus of archaea and eukarya, such as the presence of very divergent DNA polymerases [46]. However, two more recent findings suggest that LAECA already had a DNA genome: (1) the discovery in Thaumarchaeota of a type IB DNA topoisomerase that was probably present in LAECA [19] and (2) the observation of conserved genomic contexts in archaea suggesting the existence of a regulatory mechanism coupling DNA replication and translation conserved between archaea and eukarya [138]. I thus favour now a scenario in which the RNA to DNA transition (possible mediated by viruses) occurred only twice, once in the bacterial branch and the other in the branch leading from LUCA to LAECA [137]. Importantly, a “relatively simple LUCA” with an RNA genome does not mean necessarily a “very simple” LUCA, since this organism could have harboured endomembrane systems [106], possibly spliceosomes [46], and encoded a rather large amount of proteins [89]. 

For me, an appealing hypothesis is that the eukaryotic trend toward increasing complexity corresponds in fact to the continuation of the major trend that operated from the origin of life up to modern organisms, via LUCA and LAECA, whereas archaea and bacteria, far from being intermediate “primitive” forms, originated by a reversal of this trend (Figure 3). Importantly, Gouy and colleagues have shown that, similarly to LACA, *the last bacterial common ancestor* (LBCA) was probably also a thermophile [80, 81]. If the universal tree is indeed rooted in the bacterial branch, this implies (LUCA being a mesophile) that adaptation to thermophily has occurred twice independently, once in the branch leading to archaea and once in the branch leading to bacteria (Figure 3). I wonder if the fact that archaea and bacteria experienced similar selection pressure at their origin (adaptation to high temperature) could explain why they share partly similar types of mobile elements? As for archaea, bacteria might have escaped most RNA viruses (not all in that case) and retroviruses by “thermoelimination”. 

In the case of bacteria, an important event in the formation of this lineage was the invention of peptidoglycan and thick cell walls. The wide distribution of genes involved in the biosynthesis of peptidoglycan in bacterial genomes [139] suggests that this unique structure was already present in the LBCA. This invention could have dramatically reduced the number of viral lineages effective against bacteria, allowing bacteria to escape those lineages of viruses that are now archaea specific [140–142]. The efficiency of peptidoglycan against some devices produced by archaeal viruses is well illustrated by the failure of archaeal virus-associated pyramids (VAP) expressed in *Escherichia coli* to cross the peptidoglycan [143]. Archaeal VAP accumulate in the periplasm of *E. coli*, whereas those expressed in *Sulfolobus solfataricus* are formed and exposed at cell surface where they open for virions egress [143]. 

## 11. An Archaeon Is Born 

To conclude this paper, let us have a time vision back to a population of LAECA-like organisms, relatively complex cells with internal membranes and spliced genes, infected by a myriad of diverse DNA and RNA viruses. In that population, a particular bug has two offsprings, each of them gave rise to many lineages by binary fission. In one of these lineages, cells improved their capacity for phagocytosis, increased their size, and became first class predators (the ancestors of eukaryotes). Some of them invent the nucleus and become free from many viruses that infected LAECA, except those that, in a first time, could replicate in the cytoplasm (later on, some viruses will find their way to the nucleus). To escape these big raptors [50], cells from another lineage started to reduce their size and increased their growth rate. Among their descendants, a particular lineage survives all damn big raptors living around by jumping into hot water. In that process, they get rid of many viruses that tortured them before (in particular all RNA viruses), but many viruses succeeded to follow them. Some descendants of these first hot swimmers started to like it very hot; making use of isoprenoids, they built a new type of membrane, and, fusing a helicase and a topoisomerase, they invented an amazing enzyme, reverse gyrase, to stabilize (we still do not know how) their genomes [144]. These superbugs became the only organisms capable of living at temperature near (or above) the boiling point of water. One of them became LACA, the last common ancestor of all modern archaea, organisms that had become so sophisticated in their way of life and physiology that they are now capable of confronting the giant descendants of the big raptors, sometimes even to live inside their guts.

At some point in that story, either before or after the emergence of LACA, archaea and/or protoarchaea have met other microbes in hot springs, bacteria. These fast-growing microbes also had succeeded to escape predators for a while and to get rid of many viruses previously disturbing their ancestors by inventing peptidoglycan (bringing with them some viruses well known by archaea), but they have not invented a new type of membrane. They have just adjusted their classical version to better survive in hot water. They have not invented reverse gyrase, but many of them will capture this amazing enzyme from archaea to thrive happily in hot water [145]. However, bacteria have invented another enzyme, DNA gyrase, which provides them with a dramatic selective advantage by coupling gene expression rapidly to environmental fluctuations via supercoiling-dependent modification of promoter activities [146]. With peptidoglycan as armour and gyrase to adapt rapidly changing environments, bacteria were ready to rule the world; they have now invaded all biotopes in the air, soil, and sea (except when temperature exceed 95°C) and the body of all organisms larger than themselves. However, archaea will survive this bacterial expansion and expand themselves out of their initial hot cradle. Taking benefit of their unique lipids, they will thrive in energy poor biotopes, deep in the ocean, or in soils and lakes with low oxygen content [147]. Later on, catching gyrase from bacteria, some euryarchaea (Haloarchaea, Archaeoglobales, Thermoplasmatales, and Methanogens) will become able to confront and coexist with bacteria with equal efficiency in many different types of environments [148]. 

## 12. Conclusion

The Scenario I favoured in this paper for the origin and evolution of archaea is at odds with the traditional view that “prokaryotes” gave rise to “eukaryotes”. This traditional paradigm is so entrenched in our minds that it is not surprising that so many scientists endorse now “*fusion scenarios”* or “*archaeal ancestor's scenarios”* despite their many weaknesses. The confusing view that prokaryotes (assimilated to archaea and bacteria) predated eukaryotes (assimilated to modern eukaryotes) is inherent to the nomenclature “prokaryotes”, meaning “*before the nucleus”*. This is only one of the drawbacks of using the term prokaryote. I agree on this point with Pace who has strongly advocated to completely repel the term prokaryote [149]. However, despite the work of Woese and his followers, the unfortunate term prokaryote is still widely used for its convenience and I use it myself in this paper (although between “brackets”). In some cases, indeed, it is useful to refer to archaea and bacteria as two groups sharing similar traits (the coupling of transcription and translation, for instance) that are characteristic of the “prokaryotic” phenotype. In the future, I will try to replace the term prokaryote by the neutral term “akaryote”, meaning without nucleus, that I proposed twenty years ago [150], a term that was reused recently by Harish and colleagues [91]. I proposed in the same 1992 paper to rename in parallel eukaryotes by the neutral term synkaryotes (with a nucleus). Indeed, I think today that it will be very difficult to get rid of the term “prokaryote” as long as we will use the term “eukaryote”. However, synkaryote, referring to a phenotypic trait, is not really adequate to name a domain, defined instead by genotypic traits [53]. At the moment, I am favouring the name *Splicea*, instead of eukarya, since possession of the spliceosome is a unique common trait to all “eukaryotes” derived from LECA. With this name, LECA becomes the LSCA and LAECA the LASCA, why not? Indeed, the origin (and fate) of the spliceosome(s) is, in my opinion, one of the more important questions in the history of life. If you like this novel nomenclature, you can change eukarya by Splicea and eukaryotes by spliceotes in this text, LECA by LSCA, and LAECA by LASCA and read it again with a fresh mind. The proposed hypotheses will possibly then seem less unorthodox to you.
